# Goat Uterine DBA^+^ Leukocytes Differentiation and Cytokines Expression Respond Differently to Cloned versus Fertilized Embryos

**DOI:** 10.1371/journal.pone.0116649

**Published:** 2015-01-28

**Authors:** Lijuan Qin, Mingzhu Lei, Dandan Zhao, Aihua Wang, Yaping Jin, Xuefeng Qi

**Affiliations:** College of Veterinary Medicine of Northwest A & F University, Yangling, 712100, China; GI Lab, UNITED STATES

## Abstract

High rate of fetal mortality in ruminant somatic cell nuclear transfer (SCNT) pregnancies is due, at least in part, to immune-mediated abortion of fetuses. In the present study, goat uterine leukocytes were isolated by *Dolichos biflorus* agglutinin (DBA) coated magnetic beads, and with majority being were CD56^+^CD16^-^ in phenotype with low levels of perforin and Granzyme B expression. The responses of the isolated cells to SCNT and *in vitro* fertilization (IVF) embryos conditioned mediums containing hormone steroids were compared by measuring their phenotype and cytokines expression. The results showed there was a 2-fold increase in the numbers of isolated uterine leukocytes after incubation with different conditioned mediums for 120 h. However, significantly lower percentage and absolute numbers of uterine CD56^+^CD16^-^ leukocytes incubated with SCNT conditioned mediums were detected as compared with those incubated with IVF conditioned mediums (P < 0.05). The group treated with progesterone (P_4_) or the combination of P_4_ and 17β-estradiol (E_2_) were associated with significantly higher percentage and absolute numbers of CD56^+^CD16^-^ cells as compared with those treated with E_2_ alone (P < 0.05). Furthermore, in the presence of steroids, the isolated leukocytes incubated with SCNT conditioned mediums associated with greater levels of IFN-γ secretion and expression, as well as lesser levels of VEGF, as compared with those treated with IVF conditioned mediums (P < 0.05). In conclusion, this study demonstrates that SCNT embryos have a profound effect on the phenotype expression of goat uterine DBA^+^ leukocytes, as well as the secretion and expression of IFN-γ and VEGF by these cells *in vitro*.

## Introduction

The ability to produce clones by somatic cell nuclear transfer (SCNT) has improved significantly in the last decade. However, the first trimester losses of more than 50% of the transferred embryos are common for SCNT pregnancies in cattle, sheep, and goats [1, 2]. It appears that the majority of fetal rejection in SCNT pregnancies in ruminants is due to immune-mediated placental rejection [3, 4]. Limited studies in goats have shown that the reproductive tract is a site rich in lymphocytes in the secretory phase of the cycle, and increasing numbers of NK-like cells were found in the endometrium during early pregnancy [5]. In humans and mice, uterine natural killer (uNK) cells represent a distinct lymphocyte subset with a central role in immune regulation during pregnancy. The CD56^bright^CD16^-^ uNK cells are lowly cytotoxic and are the primary source of NK-derived immunoregulatory cytokines, such as IFN-γ and VEGF [6]. Regulation of immune cells in the uterus during pregnancy has been demonstrated in ruminants [7]. However, the role of embryo in modulating the differentiation and secretive activity of uterine leukocytes in ruminants remains unclear. In addition, previous study showed that the numbers of lymphocytes in the goat endometrium are influenced by the hormonal environment during early pregnancy [8]. An inadequate uterine environment induced by insufficient steroid concentration during early pregnancy in ewes can cause early embryonic death [9].

The main goal of the present study was to compare the responses of goat uterine NK-like cells to SCNT embryos vs. *in vitro* fertilization (IVF) embryos under the effects of two major ovarian steroids, progesterone (P_4_) and 17β-estradiol (E_2_). The effects of conditioned mediums derived from an *in vitro* goat embryo-endometrium co-culture system on these cells were investigated by measuring their phenotype and cytokines expression.

## Materials and Methods

### Ethics Statement

All experiments were approved by Care and Use of Animals Center, Northwest A&F University. This study was carried out in strict accordance with the Guidelines for the Care and Use of Animals of Northwest A&F University. Goat’s endometrial tissues were collected from Yangling Keyuan Cloning Co., Ltd (Yangling, China). Every effort was made to minimize animal pain, suffering and distress and to reduce the number of animal used.

### Production of SCNT and IVF embryos

SCNT and IVF procedures were performed as described by Zhang et al. [10]. Ovaries were collected from a local abattoir and transported to the laboratory in 0.9% NaCl. Cumulus- oocyte complexes (COCs) were recovered (from follicles 2 to 6 mm in diameter) by cutting with a scalpel. The COCs were washed three times and then cultured in TCM-199 (Gibco-BRL, Grand Island, NY, USA) supplemented with 10% fetal bovine serum (FBS), 0.075 U/ml human menopausal gonadotropin (Livzon, Ningbo, China), 1μg/ml E_2_, 10 ng/ml epidermal growth factor (EGF), 1% (v:v) insulin-transferrin-selenium (ITS), and 0.2 mmol/l sodium pyruvate for 22–24 at 38.5°C under an atmosphere of 5% CO_2_ in air. Oocytes with the first polar body were selected for enucleation after 24 h of *in vitro* maturation. Both the polar body and metaphase plate were removed, and then a single round donor was injected into the perivitelline space of the enucleated oocyte. To exclude specific effects of a particular donor cell culture, SCNT embryos were reconstituted with the cell nuclei of fibroblast cells originating from different goat fetuses at Day 40 of gestation from a Saanen dairy goat (Yangling Keyuan Biotechnology Inc., Yangling, China). IVF embryos were produced by fertilization of oocytes with semen derived from a single Saanen buck (Yangling Keyuan Biotechnology Inc., Yangling, China). Thus, the genetic variability in the SCNT embryos can be assumed to be similar to that in the IVF group of embryos. After SCNT or IVF, embryos were cultured under identical conditions [10] to the blastocyst stage (Day 7). Briefly, presumptive embryos were cultured in 400 μl droplets of mSOF medium (Sigma, St. Louis, MO) covered with mineral oil at 38.5°C and 100% humidity.

### Isolation and primary culture of endometrial cells

Primary goat endometrial epithelial cells (EECs) and stromal cells (ESCs) were isolated from the endometrium of 8-month-old goats (n = 6) on Day 7 of estrous cycle as previously described [11]. The primary endometrial cells were cultured and maintained in DMEM/F-12 (Gibco, Invitrogen Corporation, Grand Island, NY) supplemented with 10% charcoal stripped fetal calf serum (FCS; Biowest, France), antibiotics (100 U/ml penicillin, 100 μg/ml streptomycin; Gibco) at 37°C in a 5% CO_2_ –95% air atmosphere. The primary endometrial cells cultured on the cover slides were immediately fixed in 95% ethanol (v/v) and immunostained for cytokeratin and vimentin using an avidin-biotin complex (ABC) immunoperoxidase kit (Vectastatin Elite ABC kit; Vector Labs, Burlingame, CA) [11]. The protein expressions of 17β-estradiol receptor (ER) and progesterone receptor (PR) were analyzed separately in the EECs and ESCs using western blotting as previously described [12].

### Development of co-culture system and preparation for the conditioned medium

In experiments involving the co-culture system of embyros and endometrial cells, EECs and ESCs were grown separately to confluence on 0.4-micron-pore-size/6.4-mm-diameter Falcon cell culture insert (Fisher Scientific, Pittsburgh, PA) coated with Matrigel (without phenol red; Collaborative Biomedical Products, Bedford, MA) and in 24-well plates, respectively. Once epithelial and stromal cells were grown separately to confluence, mediums were removed from the apical and basolateral compartments and replaced with control medium or medium containing E_2_ (10^-9^ M, Sigma-Aldrich), P_4_ (10^-7^ M, Sigma-Aldrich), or E_2_ + P_4_ (10^-9^/10^-7^ M). Inserts of polarized epithelial cells were then transferred to 24-well plates containing stromal cells. The SCNT-derived or IVF-derived blastocysts were transferred unselectively into confluent epithelial cell monolayer grown on inserts with an average of two SCNT or IVF embryos per insert. After co-cultures were incubated undisturbed at 37°C in a 5% CO_2_ humidified chamber for 72 h, the supernatants (so called conditioned medium) in basolateral compartments were collected. When the control medium was prepared, the basic culture medium was added to above co-cultured system without steroids or embryos. The medium was collected after 72 h and used as the control medium. The conditioned medium and control medium were centrifuged (10,000 × *g*, 5 min), passed through a 0.22-μm filter (Millpore, Billerica, MA), and stored at-80°C until assayed.

### Isolation and evaluation of uterine leukocytes

Uterine leukocytes were isolated by *Dolichos biflorus* agglutinin (DBA) coated magnetic beads. To verify the existence of DBA^+^ and CD56^+^ leukocytes subsets in the goat endometrium, the percentages of DBA^+^ and CD56^+^ cells among total endometrial leukocytes (CD45^+^) in the goat non-pregnant endometrium were analyzed by flow cytometry. Lymphocyte gating was performed based on the forward and side scatter pattern. The total endometrial leukocytes were isolated from non-pregnant goat endometrial tissues by density gradient centrifugation procedures [8]. Antibody labelling was performed as previously reported [13]. Phycoerythrin (PE)-conjugated monoclonal antibody against CD45 was obtained from Dako, High Wycombe, UK. Fluorescein (FITC)-conjugated DBA lectin and anti-CD56 conjugated with Alexa-647 were obtained from Sigma, St Louis [13]. Subsequently, uterine leukocytes were isolated using streptavidin-coated biomagnetic beads (CelLection M450, Dynal, France) conjugated with biotinylated DBA lectin according to the methods developed by Bizinotto et al. [14]. Briefly, endometrial tissues were obtained from healthy non-pregnant goats on Day 1 after oocyte retrieval in the case of IVF treatment, minced with razor blades and homogenized in Hank’s solution (Sigma, St Louis, MO) supplemented with 1% BSA (Sigma, St Louis, MO) and 1,000U DNase typeⅠ(Sigma, St Louis, MO). After washing by centrifugation with Hank’s solution, DBA lectin-conjugated magnetic beads were added to the cell suspension and DBA lectin-reactive uterine leukocytes isolated with a Magnetic Particle Concentrator (MPC-2, Dynal) and counted in a Neubauer’s chamber. The beads were removed from the uterine leukocytes with 0.1 M *N*-acetyl-D-galactosamine (Gal-Nac) (Sigma, St Louis, MO). Up to 95% cell viability could be achieved with 1.5–4.5 × 10^5^ uterine DBA^+^ leukocytes recovered per goat endometrium tested by trypan blue exclusion. To confirm the purity and NK-like activity of the isolated uterine leukocytes, a total of 1.0 × 10^5^ isolated cells were prepared and stained with the following monoclonal antibodies: Alexa-647-conjugated anti-CD56 monoclonal antibodies, anti-CD16 conjugated with PE, anti-Granzyme B conjugated with FITC, and PE-conjugated anti-perforin (20 μg/ml; BD Biosciences, San Jose, CA). The ratio of CD56^+^CD16^-^, CD56^+^Granzyme B^+^, CD56^+^perforin^+^ subsets was reported as a percentage of over 1.0 × 10^5^ isolated cells. Isotype-matched negative-control antibodies labeled with FITC, PE or Alexa-647 were all from BD Biosciences. Flow cytometry staining was performed according to the manufacturer’s instructions. Samples were run on Beckman-Coulter Epics Altra flow cytometer (Beckman-Coulter, Fullerton, CA), a fluorescence-activated cell sorter (BD Biosciences), and analyzed by WinMDI (http://www.methods.info/software/flow/winmdi.html) and Flowjo softwares (Tree Star, Inc. Ashland, OR).

### Histology

To further confirm the incidence of DBA lectin positive uterine leukocytes in the non-pregnant goat endometrium, endometrium samples were prepared for histologic examination. Paraffin sections (7 µm) of endometrium were processed for DBA lectin cytochemistry according to Chen et al. [15]. Briefly, it consisted in incubated with biotinylated DBA lectin, followed by streptavidin-peroxidase (DAKO, CA) and revealed with 3,3,-diaminobenizidine (Sigma, St Louis, MO) and hydrogen peroxide reaction. The control reaction was performed by adding 0.1 M N-acetyl-D-galactosamine (NacGal) in the DBA lectin solution before the incubation with secretions.

### Flow cytometry

The changes on proportion and absolute numbers of CD56^+^CD16^-^ cells among isolated uterine leukocytes over 120-hr incubation with different conditioned mediums in comparison with the cells at the beginning of the culture period were analyzed by flow cytometry. Briefly, freshly isolated uterine leukocytes were seeded at 2 × 10^5^ cells per well on a 96-well culture plate and cultured with conditioned medium or control medium for 120 h. Cells were then prepared and stained with the designated monoclonal antibodies: Alexa-647-conjugated anti-CD56 monoclonal antibodies and anti-CD16 conjugated with PE (BD Biosciences, San Jose, CA). Mouse serum was used to block non-specific Fc-receptor binding, and homologous IgGs were used as negative control antibodies. In addition, the effects of conditioned medium on proliferation of isolated uterine leukocytes were determined using a bromodeoxyuridine (BrdU) cell-proliferation assay as previously described [16].

### Cytokine analysis by enzyme-linked immunosorbent assay (ELISA)

Isolated uterine leukocytes were adjusted in the conditioned medium at the concentration of 1 × 10^6^ cells/ml. Two hundred microliters (2 × 10^5^ cells) of suspension was cultured in 96-well culture plate at 37°C in a humidified 5% CO_2_ incubator for 120 h. The supernatants collected from 96-well culture plate were assayed using ELISA kit (Sunma, Xiamen, China) developed specifically for caprine cytokines [11, 17].

### Western blot analysis

We also determined the protein expression of IFN-γ and VEGF in isolated uterine leukocytes incubated with conditioned medium for 120 h. The cell lysates were prepared in lysis buffer (10 mM Tris-HCl, 50 mM NaCl, 5 mM EDTA, 50 mM NaF, 0.1 mM sodium orthovanadate, 30 mM sodium pyrophosphate, 1% Triton X-100, 10 μg/ml leupeptin, and 10 μg/ml aprotinin) on ice for 30 min. The cell lysates were separated on 12% SDS-polyacrylamide gels and blotted onto polyvinylidene difluoride membranes and probed with mouse monoclonal antibodies against goat IFN-γ and VEGF (Abcam Systems, Cambridge, MA), respectively. The bands were visualized using HRP-conjugated goat anti-mouse IgG (Vector Laboratories Inc., Burlingame, CA) followed by ECL protocol (Amersham Biosciences, Little Chalfont Buckinghamshire, UK). The protein bands were recorded using a Konica SRX 101A developer (Konica Minolta Medical Imaging). Densitometric analyses were performed (protein of interest and β-actin) with the GelDoc 2000 and the Quantity one software (Bio-Rad, Mississauga, ON, Canada).

### Statistical analysis

All experiments shown were performed a minimum of five times, and the data were calculated as the mean ± standard error of the mean (SEM). The effects of steroids on all dependent measures were analyzed with two-way analysis of variance (ANOVA) with treatment group and/or embryos as the independent variable(s), using SPSS v19.0 (SPSS Inc., USA). Comparisons among leukocytes over 120-hr incubation with different conditioned mediums or control medium were conducted with Student t-tests. Significant interactions were further analyzed using the Tukey method for pairwise multiple comparisons. A *P*-value of less than 0.05 was regarded as statistically significant. The data shown in some figures (e.g. photographs of flow cytometry analysis) were from a representative experiment, which was qualitatively replicated in at least five independent experiments.

## Results

### Cell morphology and detection of steroid receptor

**Figure 1:**
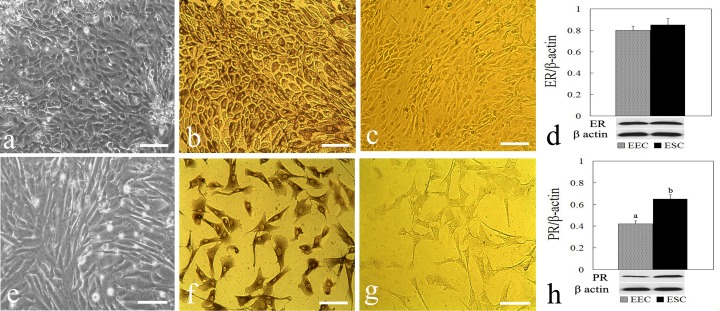
Characterization of primary endometrial epithelial cells (EECs) and endometrial stromal cells (ESCs). The cobblestone structure and expression of cytokeratin were shown in EECs (a, b) and the spindle-shape structure and vimentin expression were shown in ESCs (e, f). The negative control shown of cytokeratin and vimentin involved use of mouse IgG in place of primary antibody (c, g). Western blot analysis of estrogen receptor (ER) and progesterone receptor (PR) protein expression were shown separately in the endometrial cells (d, h). Results were quantified by densitometry analysis of the bands and normalization to β-actin protein. Data represent the mean ± standard error of the mean (SEM) from five independent experiments. Columns with different superscript letters are significantly different (*P* < 0.05). Scale bar = 30 µm.

After 72 h to 96 h of culture, both the EECs and ESCs reached subconfluent monolayer that showed polyhedral-shaped and fibroblast-like appearance (Fig. 1a, e), respectively. The EECs were stained by the epithelium-specific cytokeratin antibody (Fig. 1b), whereas the ESCs exhibited vimentin staining (Fig. 1f). Western blot analysis showed that both ER and PR proteins were detected in the EECs and ESCs. No significant differences were observed in the ER expression between EECs and ESCs (Fig. 1d), while the PR expression abundance in ESCs was significantly greater than that in EECs (*P* < 0.05, Fig. 1h).

### Blastocyst attachment and outgrowth on the endometrial epithelial cell monolayer

**Figure 2:**
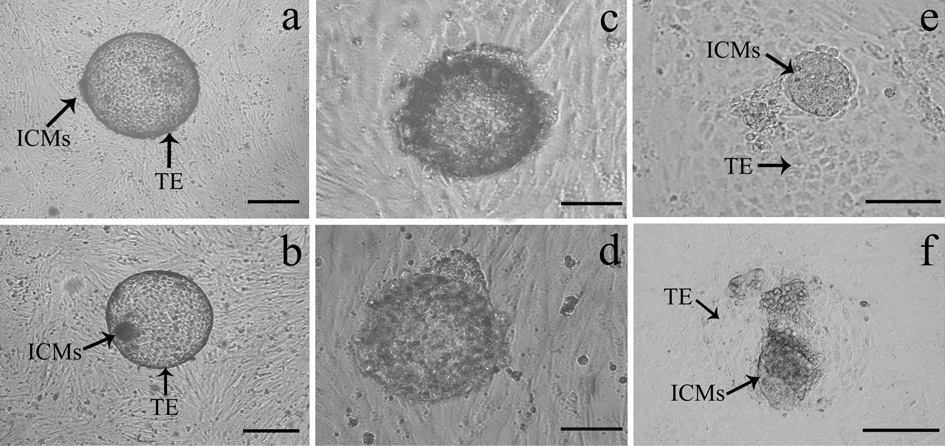
Different developmental stages of goat embryos produced *in vitro*. Transferred IVF blastocysts (a) or SCNT blastocysts (b) showed clear inner cell masses (ICMs) and trphoectodermal cells (TE) under bright field at Day 7. Both the IVF blastocysts (c) and SCNT blastocysts (d) attached to the endometrial epithelial cells after co-cultured for 24 h. The trophoblast of IVF (e) and SCNT (f) underwent outgrowth on the epithelial cells monolayer after co-cultured for 72 h. Scale bar = 50 µm.

In the initial experiment, two hatched SCNT blastocysts, or IVF blastocysts, were randomly allocated to per insert well containing confluent endometrial epithelial cells. Nearly all the IVF or SCNT blastocysts transferred showed clear inner-cell-mass (ICM) and trphoectodermal cells (TE) under bright field at Day 7 of culture (Fig. 2a, b). Both the IVF and SCNT blastocysts began to adhere to endometrial epithelial cells after co-culture for 24 h (Fig. 2c, d), and the trophoblast of IVF and SCNT underwent outgrowth on the epithelial cell monolayer after co-culture for 72 h (Fig. 2e, f). Leaving the undisturbed co-culture for 96 h did not lead to any further increase compared with attachment at 72 h incubation. Hence, in later experiments, we incubated IVF or SCNT blastocysts with EECs-ESCs system without observation for 72 h to collect conditioned mediums from the apical chambers.

### Isolation of uterine leukocytes and phenotypic analysis

**Figure 3:**
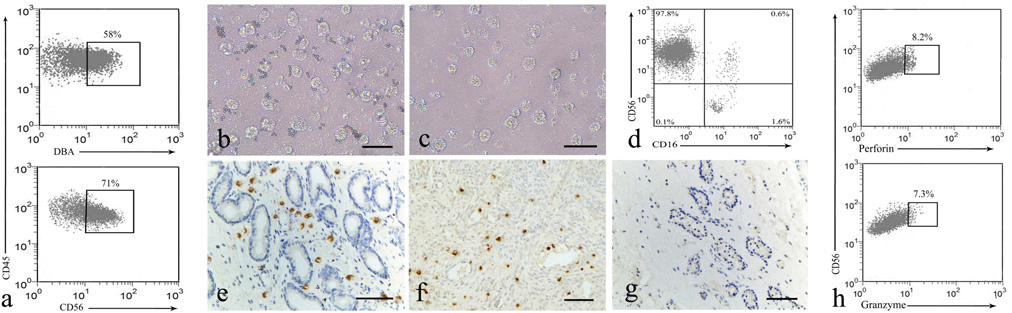
Isolation and identification of uterine *Dolichos biflorus* agglutinin (DBA) positive leukocytes in non-pregnant goat endometrium. Lymphocytes were identified by their size and granularity in the FSC/SSC dot plot. Representative flow cytometry analysis of the percentages of DBA^+^ and CD56^+^ cells among CD45^+^ leukocytes in the non-pregnant goat endometrium (a). Differential interference contrast (DIC) image of viable goat uterine leukocytes attached to DBA^+^ lectin-coated magnetic beads (b) and detached after addition of 0.1 M Gal-NAc (c). The expression of CD56, CD16 on the isolated uterine leukocytes was evaluated by flow cytometer (d). Representative microphotographs for DBA lectin reactive uterine leukocytes scattered between endometrial glands (e) and stroma (f) of the non-pregnant goat endometrium. No positive reaction was seen in the control reaction performed with DBA lectin inhibited with NacGal (g). Representative flow cytometry analysis of the expression of perforin and Granzyme B on gated CD56^+^DBA^+^ leukocytes isolated (h). Scale bar = 30 µm (b, c) and 90 µm (e-g).

As shown in Fig. 3a, the percentages of DBA^+^ and CD56^+^ cells among CD45^+^ leukocytes in the non-pregnant goat endometrium was 56% ± 2.13% and 68% ± 3.35%, respectively. By using DBA lectin-conjugated magnetic beads, viable and highly purified goat uterine leukocytes were effectively isolated (Fig. 3b, c). The expressions of CD56, CD16, perforin and Granzyme B on the purified cells were evaluated by flow cytometer. The purity of CD56^+^CD16^-^ cells was routinely more than 95.32% ± 1.86% (Fig. 3d). The expressions of CD56^+^perforin^+^ and CD56^+^Granzyme B^+^ on the isolated cells were 6.21% ± 1.35% and 7.3% ± 1.66%, respectively (Fig. 3h).

### Isolated uterine leukocytes location in the endometrium

The DBA lectin cytochemistry selectively labeled the cell surface and granules of uterine leukocytes accumulated between the endometrial glands and distributed throughout the stroma of the non-pregnant goat endometrium (Fig. 3e, f). No positive reaction was seen in the control reaction performed with DBA lectin inhibited with NacGal (Fig. 3g).

### Isolated uterine leukocytes phenotype in response to conditioned medium

**Figure 4:**
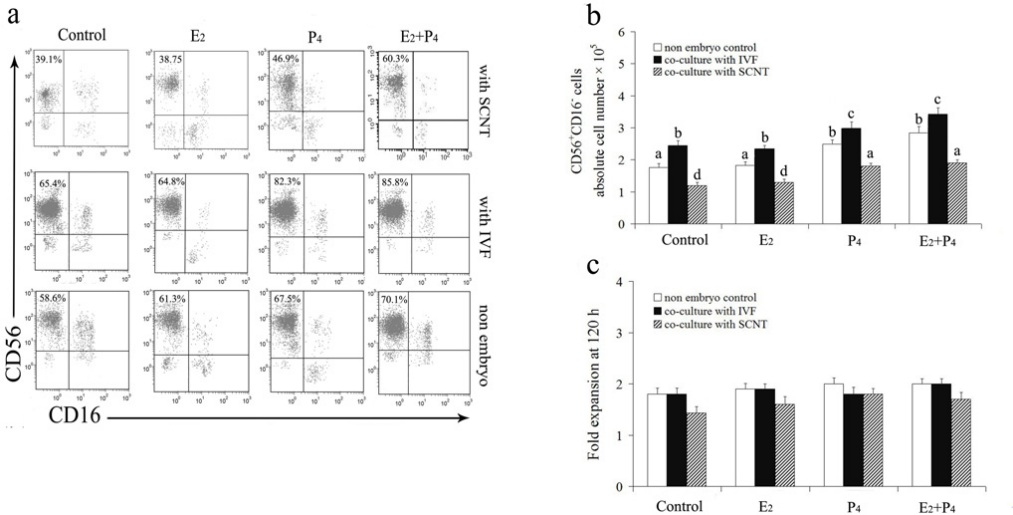
Flow cytometry analysis of the expression of CD56^+^CD16^-^ on goat uterine leukocytes. Representative flow cytometry analysis of the percentage of CD56^+^CD16^-^ cells on isolated goat uterine leukocytes in response to cloned and fertilized embryos (a). The absolute numbers of uterine CD56^+^CD16^-^ leukocytes over 120-hr incubation with different conditioned mediums or control medium (b). Fold expansion of total numbers of isolated goat uterine leukocytes after 120-hr incubation with different conditioned mediums or control medium in comparison to the cells at the beginning of the culture period (c). *Columns* and *vertical bars* represent the mean ± SEM from at least five independent experiments, and analyzed by two-way ANOVA with treatment group and/or embryos as the independent variable(s). Columns with different superscript letters are significantly different from each other (*P* < 0.05).

Under the treatment of P_4_ or the combination of P_4_ and E_2_, both the percentage and absolute numbers of CD56^+^CD16^-^ cells were significantly greater as compared with those treated with E_2_ alone regardless of incubation with conditioned medium or control medium (*P* < 0.05, Fig. 4a, b). Interestingly, the percentage and numbers of CD56^+^CD16^-^ leukocytes incubated with SCNT conditioned medium were significantly lesser in contrast with those incubated with IVF conditioned medium or control medium in the presence of steroids or not (*P* < 0.05, Fig. 4a, b). In addition, proliferation was increased in isolated uterine leukocytes (DBA^+^) over 120-hr incubation with conditioned medium or control medium in comparison to the cells before the culture period, but the results did not reach statistical significance (Fig. 4c). There is no significant difference of the total numbers of isolated leukocytes between over 120-hr incubation with different conditioned mediums and control medium (data not shown).

### Isolated uterine leukocytes cytokines secretion and expression in response to different conditioned mediums

**Figure 5:**
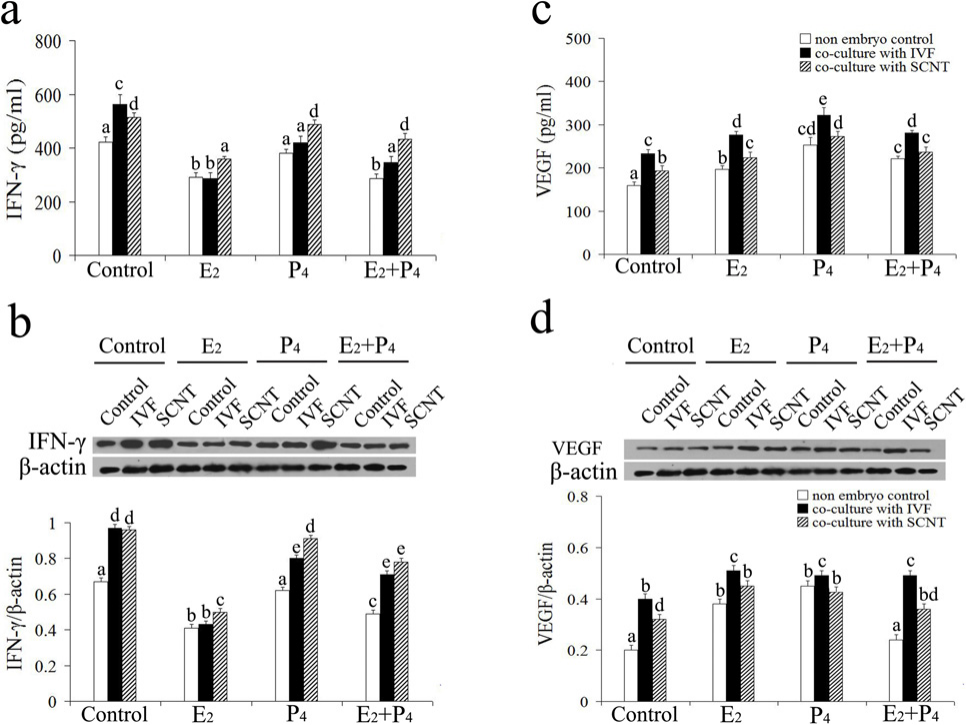
The secretion and expression of cytokines by uterine DBA^+^ leukocytes in response to embryos and steroids. ELISA analyses of the concentrations of IFN-γ (a) and VEGF (c) secreted by uterine DBA^+^ leukocytes in response to cloned and fertilized embryos. Representative western blotting and densitometric analysis of IFN-γ (b) and VEGF (d) normalized to β-actin to correct for protein loading. *Columns* and *vertical bars* represent the mean ± SEM from at least five independent experiments, and analyzed by two-way ANOVA with treatment group and/or embryos as the independent variable(s). Columns with different superscript letters are significantly different from each other (*P* < 0.05).

Overall, IVF or SCNT conditioned medium stimulated secretion of IFN-γ and VEGF by isolated uterine leukocytes in the presence of steroids or not. The levels of IFN-γ in the cell supernates incubated with SCNT conditioned medium were significantly greater than those co-cultured with IVF conditioned medium under the treatment of P_4_ and/or E_2_ (*P* < 0.05, Fig. 5a). However, the VEGF levels in the supernate of cells incubated with SCNT conditioned medium were significantly lesser than those for incubated with IVF conditioned medium in the presence of steroids (*P* < 0.05, Fig. 5b). Overall, the levels of IFN-γ and VEGF in the supernates of uterine leukocytes treated with P_4_ alone or the combination of P_4_ and E_2_ were significantly greater than those treated with E_2_ alone (*P* < 0.05, Fig. 5a, b). Western blot analysis revealed that IFN-γ and VEGF antibody recognized a protein which migrated at 17 kDa and 40 kDa, respectively (data not shown). Densitometric analysis of cytokines expression showed that the protein abundance of IFN-γ and VEGF in the cells incubated with different conditioned mediums was in accordance with secreted concentration as demonstrated by ELISA analysis (*P* < 0.05, Fig. 5c, d).

## Discussion

In the present study, IVF embryos were used as a control for the identification of specific responses of the uterine DBA^+^ leukocytes derived from non-pregnant endometrium to SCNT embryos. The IVF protocol used in this study had been previously shown to produce fetuses that are not different from *in vivo*-derived fetuses in growth and endocrine parameters [18]. DBA lectin reactivity has been widely used for histological recognition and isolation of uterine NK cells in pregnant mice [14, 15]. Furthermore, CD56 and CD16 were normally used as molecular evidence for the identification of NK-like cells based upon their expression patterns in peripheral and uterine NK cells of other species [13, 19]. The present study provides the first evidence for goat uterine leukocytes sharing both DBA lectin positive and CD56^+^ CD16^-^ phenotype by using flow cytometry and cytochemistry analysis. The expression of DBA and CD56 on goat uterine leukocytes seen here are particularly interesting because to date, expression of these molecules on the uterine leukocyte has only been observed in humans and mice [14, 19]. In most species, NK cells of the uterine and decidua are distinct from peripheral populations in terms of phenotypes and functions [20]. Despite the implications of their name, uterine NK-like cells are weakly lytic and promote the establishment and maintenance of pregnancy [16, 21, 22]. Our data also showed weak expression of perforin and Granzyme B on isolated goat uterine leukocytes.

Pregnancy in ruminants results in a change in numbers and functions of immune cells in uterus that potentially affects fetal survival and uterine defense mechanisms postpartum [9]. These changes are driven by local signals from the conceptus as well as from hormonal changes mediated by the placenta or maternal system [7]. There is increasing evidence for immunological cause of pregnancy loss associated with cloned fetuses in ruminants [4, 23]. The present study demonstrates that there are differences in the phenotype and cytokines expression of goat uterine leukocytes in response to cloned versus fertilized embryos. Although similar fold of proliferation of isolate over 120-hr incubation with different conditioned mediums or control medium was detected, they differed in their phenotype expression. Our data showed significantly decreased percentages and absolute numbers of uterine CD56^+^CD16^-^ leukocytes incubated with SCNT conditioned medium as compared with those incubated with IVF conditioned medium. In general, abnormal placental development and associated consequences for embryo-maternal communication during the early pregnancy have been a main limiting factor in ruminant SCNT-derived pregnancies [24]. The gene expression profiles in endometrium samples from SCNT pregnant cows were different from those from IVF-derived pregnancies [4]. In addition, transforming growth factor superfamily produced by endometrial stromal cells and trophoblasts also plays a key role in transforming uterine leukocytes into a tolerant-immunosuppressive phenotype *in vitro* [25, 26]. It appears that the abnormal phenotype expression of the isolated uterine leukocytes co-cultured with SCNT conditioned medium is due to different expressions of the cytokines and modulator by endometrial cells in response to SCNT embryos. Furthermore, our data showed that the groups treated with P_4_ or the combination of P_4_ and E_2_ were associated with significantly greater percentage and absolute numbers of CD56^+^CD16^-^ cells as compared with those treated with E_2_ alone. Previous studies demonstrated that most genes in the bovine endometrium were stimulated by circulating concentrations of P_4_ during early gestation, and these modulations have direct effects on the differentiation and accumulation of lymphocytes in the endometrium [9, 27, 28].

It is interesting to note that, under the treatment of hormone steroids, the isolated uterine lymphocytes incubated with SCNT conditioned medium were associated with greater levels of IFN-γ secretion and expression, as well as lesser levels of VEGF, as compared to those for cells incubated with IVF conditioned medium. Similar to the present study, a striking increase of pro-inflammatory cytokine levels was detected in endometrial flushing from SCNT pregnant cows [3]. This may attribute to abnormal nonclassical, major histocompatibility complex (MHC) class I antigens expression on SCNT embryos at the earliest stage of pregnancy in ruminants [3]. Constant natural killer cell receptor (NKR)-MHC interactions fine-tune NK responsiveness to match the MHC environment so that NK cells remain tolerant to self, yet responsive [29, 30]. Recently study in mice suggests that MHC molecules can influence uterine NK cell maturation and directly inhibit or activate uterine NK cell function [31]. Normally, suppression of MHC class I during the first trimester of ruminant pregnancies is temporally associated with the initiation of intimate contact between endometrium and the chorioallantois. However, the majority of SCNT conceptuses expressing MHC class I antigens at the very earliest stages of pregnancy are associated with a pronounced endometrial lymphocytic responses [32].

Very little is known so far on the effects of SCNT embryos on goat uterine leukocytes VEGF expression, although it has been shown that the VEGF system seems to be disturbed in placentomes of cloned bovine fetuses [33]. Our *in vitro* findings corroborated the hypothesized role of the VEGF system for placental vasculogenesis and angiogenesis, which seems to be disturbed in placentomes of cloned fetuses [34]. Although the conditioned or control medium may be contaminated by endometrium stimulated by steroids to produce VEGF [35], western blotting analysis of the uterine leukocyte activity on VEGF synthesis suggests the distinct effects of SCNT and IVF embryos on uterine leukocytes VEGF expression.

In conclusion, a complex network of hormones, cytokines, and regulatory factors in response to SCNT or IVF embryos may contribute to different phenotypes and cytokines expression of proliferating goat uterine leukocytes *in vitro*. Nonetheless, it is clear that much more needs to be learned about the molecular mechanisms to elucidate the origin and the potential preventive treatment for SCNT-derived placental defects and to enable a better gestational outcome for cloned animals.
